# Single Nucleotide Polymorphisms Other than Factor V Leiden Are Associated with Coagulopathy and Osteonecrosis of the Femoral Head in Chinese Patients

**DOI:** 10.1371/journal.pone.0104461

**Published:** 2014-08-13

**Authors:** Kou-Ti Peng, Kuo-Chin Huang, Tsan-Wen Huang, Yun-Shien Lee, Wei-Hsiu Hsu, Robert W. W. Hsu, Steve W. N. Ueng, Mel S. Lee

**Affiliations:** 1 Department of Orthopaedic Surgery, Chang Gung Memorial Hospital, Chiayi, Taiwan; 2 Department of Biotechnology, Ming-Chuan University, Taoyuan, Taiwan; 3 Genomic Medicine Research Core Laboratory, Chang Gung Memorial Hospital, Taiwan; 4 Department of Orthopedic Surgery, Chang Gung Memorial Hospital, Linkou, Taiwan; 5 Department of Medicine, College of Medicine, Chang Gung University, Taoyuan, Taiwan; Morehouse School of Medicine, United States of America

## Abstract

Single nucleotide polymorphisms (SNPs) of factor V Leiden have been associated with osteonecrosis of the femoral head (ONFH) in Caucasians but remains controversial in Asians. We used an SNP microarray to screen 55 loci of factor V gene in patients with ONFH of Chinese. Significantly different candidate SNPs at 14 loci were analyzed in 146 patients and 116 healthy controls using MALDI-TOF (matrix-assisted laser desorption/ionization time-of-flight) mass spectrometry and gene sequencing. The factor V Leiden (rs6025) was not found in all participants. Six SNP loci (rs9332595, rs6020, rs9332647, rs3766110, rs10919186, and rs12040141) were confirmed with significant differences in patients but not in controls. The rs6020 G-to-A polymorphism was found in 88.9% of the patients. In addition, a high percentage (87.6%) of the patients had an abnormal coagulation profile that included hyperfibrinogen, elevated fibrinogen degradation products, elevated D-dimer, abnormal protein S, abnormal protein C, or a decrease in anti-thrombin III. Patients with the rs6020 G-to-A polymorphism (mutation) had a higher risk (odds ratio: 4.62; 95% confidence interval: 1.44–14.8) of having coagulation abnormalities than did those without the mutation (wild-type) (χ^2^ *p*  =  0.006). Our findings suggested that the rs6020 polymorphism might be the genetic trait that accounts for the higher prevalence of ONFH in the Chinese population than in Westerners. Exposure to risk factors such as alcohol and steroids in patients with the rs6020 polymorphism causes coagulation abnormalities and, subsequently, thromboembolisms in the femoral head.

## Introduction

Osteonecrosis of the femoral head (ONFH) is a debilitating disease involving both hips; it normally affects younger patients [1], [2]. Cases of ONFH have been related to various risk factors, including genetic mutations [3]–[12]. Factor V Leiden, the prothrombin G20210A mutation, and the MTHFR (methylenetetrahydrofolate reductase) C677T gene polymorphism are the most common genetic risk factors predisposing Caucasians to ONFH [5]–[11]. However, the correlation in Asian patients remains controversial [12]–[15]. The conflicting results are confusing because the incidence of ONFH in the Asian population is reported to be higher than in the Caucasian population. However, factor V Leiden, prothrombin G20210A, and the MTHFR mutation are either absent or irrelevant in the Asian population [2], [12]–[18].

Because the etiologies of ONFH are miscellaneous, most genetic association studies on ONFH segregated their patients into different subgroups based on the risk factors (e.g., alcohol, steroids, and smoking). This may decrease the number of samples in the subgroup analysis and may generate false results [15]. We hypothesized that Asian patients with ONFH should have genetic polymorphisms associated with coagulation abnormalities because their incidence of ONFH is higher than that of Caucasians. We focused on the factor V gene because of its importance in the coagulation cascade. We first used a microarray to screen all single nucleotide polymorphisms (SNP) of the factor V genes and then validated the results in 146 patients with ONFH and 116 controls.

## Materials and methods

The protocol of this study was approved by the Chang-Gung Memorial Hospital Institutional Review Board (IRB94-1024B). All 146 patients (109 men, 37 women; mean age: 45 ± 11 years) included in this study between November 2005 and October 2013 signed informed consents. Patient demographics, risks factors, and history of prior surgeries for ONFH were recorded. ONFH was staged according to the ARCO system [19]. Patients with caisson disease and Perthes disease were excluded from the SNP analysis because the underlying pathogenesis is different. We also recruited 116 healthy controls (54 men, 62 women; mean age: 43 ± 19 years). The controls had no risk factors, no history of thromboembolic events, and no symptoms of hip disease. After a 12-hour fast, blood samples were drawn via venipuncture to minimize circadian influence on fibrinolytic activity. Laboratory tests of coagulation profiles–prothrombin time, partial thromboplastin time, serum fibrinogen, fibrinogen degradation product (FDP), D-dimer, protein S, protein C, and anti-thrombin III–were done.

Genomic DNA was extracted and purified from the whole blood of all the participants using a kit (QIAamp DNA Blood Midi Kit; Qiagen, Hilden, Germany). Genomic DNA from 22 patients and 82 controls was used for microarray analysis (Genome-Wide Human SNP Array 6.0; Affymetrix, Santa Clara, CA, USA). The SNP genotypes and raw data of the SNP Array 6.0 are available at NCBI Gene Expression Omnibus (GEO accession number: GSE58951). The 55 SNP loci of the factor V contained in the SNP 6.0 microarray were screened for candidate SNPs. Site-specific PCR primer sets for the candidate SNP loci were used for further validation. Genotypes of the candidate SNP loci were analyzed using MALDI-TOF-MS (matrix-assisted laser desorption ionization-time of flight-mass spectrometry) in 146 patients and 116 controls on an SNP genotyping system (Bruker, Bremen, Germany). Briefly, a polymerase chain reaction (PCR) was done in a total volume of 10 µl containing 100 ng of genomic DNA, primers (1 µM each), deoxyribonucleoside triphosphate (dNTP) (1 mM), 16 FastStart PCR buffer (50 mM Tris-HCl, 10 mM KCl, 5 mM [NH_4_]_2_SO_4_, 2 mM MgCl_2_; pH 8.3) (Roche Applied Science, Basel, Switzerland), 1 M betaine, and 1 unit of FastStart Taq DNA Polymerase (Roche Applied Science). The reaction was initiated at 95°C for 5 min, followed by 40 cycles at 95°C for 30 s, 60°C for 30 s, and 72°C for 30 s, with a final extension at 72°C for 2 min. Unincorporated dNTP and primers were removed automatically using MAPII8 (GenePure PCR Purification System; Bruker). The purified products were collected and mini-sequencing reactions were run using individual mini-sequencing primers in 20 mL of a solution containing 50 ng of the PCR product, 1 mL (10 pmol) of mini-sequencing primer, 0.5 mL of 1 mM dideoxynucleoside triphosphate (ddNTP)/dNTP mixture, 0.5 units of Thermo Sequenase DNA Polymerase (Amersham Biosciences, Piscataway, NJ, USA), and 2 mL of the reaction buffer provided by the manufacturer. The reactions were done in a multiblock thermal cycler (Thermo Hybaid, Waltham, MA, USA) with initial denaturation at 94°C for 2 min, followed by 50 cycles at 94°C for 8 s, 52°C for 8 s, and 72°C for 8 s. The reaction products were purified automatically using MAPIIA (Single-Strand DNA Binding Beads; Bruker) and analyzed using MALDI-TOF-MS. The samples were then mixed with 0.5 mL of matrix solution (50 mg/mL 3-hydropicolinic acid in a 4∶5∶1 mixture of water, acetonitrile, and 50 mg/mL diammonium citrate) and spotted onto 384-well Teflon sample plates (PerSeptive Biosystems, Framingham, MA, USA). MALDI-TOF mass spectra were acquired with a mass spectrometer (Autoflex; Bruker) and AutoXecute software (Bruker) to validate the genotype data.

The allele frequencies of the SNP loci between patient group and control group were analyzed using Chi-square test. The SPSS 17.0 for Windows (SPSS Inc., Chicago, IL, USA) was used for statistical analysis. Significance was set at p < 0.05.

## Results

Ninety-eight of the 146 patients (67.1%) had ONFH in both hips (196 hips) and the other 48 had ONFH in only one hip (total  =  244 hips). Risk factors were habitual alcohol drinking in 90 patients (61.6%), steroids in 16 patients (11%), and idiopathy in 40 patients (27.4%). The majority of the patients had idiopathic cases; only 3 patients had a family history of ONFH.

There were 14 candidate SNP loci with significant differences between patients and controls (Table 1). Factor V Leiden (rs6025) was not the candidate SNP related to ONFH. All participants had the homozygous GG genotype on the rs6025 locus. The genotypes of the 14 candidate SNPs were further analyzed using MALDI-TOF with site-specific primers in the 146 patients and 116 controls (Table 2). Genotypes of 6 SNP loci (rs9332595, rs6020, rs9332647, rs3766110, rs10919186, and rs12040141) in patients were significantly different from those in controls (Table 2), and only rs6020 was in the exon 10 region of the Factor V gene. The frequency of the rs6020 G-to-A polymorphism in patients was 88.9%. The results of allele frequencies of the 6 SNP loci, except for rs3766110, were consistent with the differences genotype frequencies in patients and controls (Table 3). The allele frequency of the C-to-G mutation of rs9332595 in patients was significantly different from that in the controls (odds ratio: 6.73; 95% CI: 4.55-9.96) (χ^2^ *p*  =  0.000). The allele frequencies of the other 3 SNP loci (rs9332647, rs10919186, and rs12040141) were also significantly different between patients and controls (odds ratios: 1.48, 1.74, and 1.54, respectively).

**Table 1:** Microarray Results of Candidate Factor V SNP.

Affymetrix Probeset No	rs No.	p-value
SNP_A-1785570	rs9332595	0.01
SNP_A-1894289	rs9332600	0.01
SNP_A-1971446	rs974793	0.02
SNP_A-1971449	rs3820060	0.03
SNP_A-1971459	rs6020	0.01
SNP_A-2168989	rs4656685	0.02
SNP_A-2313966	rs9332647	0.01
SNP_A-4258379	rs3766110	0.01
SNP_A-8304582	rs4524	0.01
SNP_A-8318587	rs6662593	0.01
SNP_A-8468549	rs9332619	0.02
SNP_A-8506930	rs10919186	0.004
SNP_A-8514971	rs9332627	0.02
SNP_A-8644570	rs12040141	0.01

**Table 2:** Genotype Frequency of Factor V in Healthy Controls and Patients with Osteonecrosis (ONFH).

	Healthy Controls (%)			Patients with ONFH (%)			
RS	Wild-type	Heterozygous	Mutation	Wild-type	Heterozygous	Mutation	p-value
9332595	62 (53.9)	23 (20.0)	30 (26.1)	4 (2.8)	52 (36.1)	88 (61.1)	0.000*
9332600	83 (72.2)	26 (22.6)	6 (5.2)	91 (63.6)	46 (32.2)	6 (4.2)	0.232
974793	82 (71.3)	27 (23.5)	6 (5.2)	88 (60.7)	52 (35.9)	5 (3.4)	0.090
3820060	77 (68.1)	29 (25.7)	7 (6.2)	84 (57.9)	55 (37.9)	6 (4.1)	0.104
6020	13 (11.4)	33 (28.9)	68 (59.6)	16 (11.0)	74 (51.0)	55 (37.9)	0.001*
4656685	82 (71.9)	26 (22.8)	6 (5.3)	88 (60.7)	52 (35.9)	5 (3.4)	0.069
9332647	64 (56.1)	38 (33.3)	12 (10.5)	59 (40.7)	69 (47.6)	17 (11.7)	0.040*
3766110	85 (73.9)	23 (20.0)	7 (6.1)	88 (61.1)	52 (36.1)	4 (2.8)	0.011*
4524	83 (72.8)	25 (21.9)	6 (5.3)	91 (63.2)	46 (31.9)	7 (4.9)	0.201
6662593	83 (72.7)	26 (22.6)	6 (5.2)	91 (63.2)	47 (32.6)	6 (4.2)	0.202
9332619	82 (71.9)	26 (22.8)	6 (5.3)	88 (61.1)	50 (34.7)	6 (4.2)	0.113
10919186	74 (64.9)	35 (30.7)	5 (4.4)	58 (40.0)	87 (60.0)	0 (0)	0.000*
9332627	83 (71.6)	27 (23.3)	6 (5.2)	89 (61.0)	51 (34.9)	6 (4.1)	0.122
12040141	65 (56.5)	38 (33.0)	12(10.4)	58 (39.7)	70 (47.9)	18 (12.3)	0.023*
6025	116 (100)	0 (0)	0 (0)	145 (100)	0 (0)	0 (0)	–

*p < 0.05.

**Table 3:** Allele Frequency of Factor V in Healthy Controls and Patients with Osteonecrosis (ONFH).

RS	Allele Type	Healthy Controls (%)	Patients with ONFH (%)	Odds ratio	95% CI	p-value
9332595	C/G	147 (63.9)/83 (36.1)	60 (20.8)/228 (79.2)	6.73	4.55–9.96	0.000*
9332600	G/A	192 (83.5)/38 (16.5)	228 (79.7)/58 (20.3)			0.306
974793	C/T	191 (83)/39 (17)	228 (78.6)/62 (21.4)			0.221
3820060	T/G	183 (81)/43 (19)	223 (76.9)/67 (23.1)			0.280
6020	A/G	169 (74.1)/59 (25.9)	184 (63.4)/106 (36.6)	1.65	1.13–2.42	0.010*
4656685	C/T	190 (83.3)/38 (16.7)	228 (78.6)/62 (21.4)			0.181
9332647	G/A	166 (72.8)/62 (27.2)	187 (64.5)/103 (35.5)	1.48	1.01–2.15	0.046*
3766110	A/C	193 (83.9)/37 (16.1)	228 (79.2)/60 (20.8)			0.176
4524	A/G	191 (83.8)/37 (16.2)	228 (79.2)/60 (20.8)			0.212
6662593	G/A	192 (83.5)/38 (16.5)	229 (79.5)/59 (20.5)			0.259
9332619	C/T	190 (83.3)/38 (16.7)	226 (78.5)/62 (21.5)			0.179
10919186	C/G	183 (80.3)/45 (19.7)	203 (70.0)/87 (30.0)	1.74	1.16–2.63	0.008*
9332627	C/T	193 (83.2)/39 (16.8)	229 (78.4)/63 (21.6)			0.184
12040141	G/A	168 (73)/62 (27)	186 (63.7)/106 (36.3)	1.54	1.06–2.25	0.024*
6025	G/A	246 (100)/0 (0)	290 (100)/0 (0)			–

CI, confidence interval.

*p < 0.05.

Because the rs6020 G-to-A polymorphism accounted for 88.9% of the patients with ONFH, the correlation of the rs6020 polymorphism with risk factors and coagulation abnormalities was further analyzed. Patients with risk factor of habitual alcohol drinking were associated with higher incidence of rs6020 polymorphism (92.3%) as compared with healthy controls. (Table 4) Coagulation profiles of fibrinogen, FDP, D-dimer, protein S, protein C, and antithrombin-III were available in 129, 129, 72, 128, 128, and 99 cases, respectively. (Table 5) Abnormalities of the coagulation profile included hyperfibrinogenemia (28.7%), elevated FDP (41.1%), elevated D-dimer (44.4%), abnormal protein S (29.7%), abnormal protein C (24.2%), and decreased antithrombin III (22.2%) in patients with ONFH. Overall, high percentage (87.6%) of the patients had coagulation abnormalities caused by fibrinogen, FDP, D-dimer, protein S, protein C, or anti-thrombin III. In patients with the rs6020 G-to-A polymorphism (mutation), the risk for coagulation abnormalities (odds ratio: 4.62; 95% CI: 1.44–14.8) was significantly (*p*  =  0.006) higher than in patients without this mutation (wild-type).

**Table 4:** Number of Patients with rs6020 Gene Polymorphism Stratified by Risk Factor.

Risk Factor	Genotype (%)			*p*-value*
	Wild-type	Heterozygous	Mutation	
Alcohol	7 (7.8)	50 (55.6)	33 (36.7)	0.001
Idiopathic	7 (18)	16 (41)	16 (41)	0.128
Steroid	2 (12.5)	8 (50)	6 (37.5)	0.202

* Compared with healthy controls.

**Table 5:** Number of Patients with rs6020 Gene Polymorphism and Coagulation Abnormalities.

	Coagulation Profile Abnormalities										
	With (%)										
	Fibrinogen	FDP	Protein S	Protein C	AT-III	D-dimer	Overall	Without (%)	Odds Ratio	95% CI	p-value
Mutation	33 (25.6)	50 (38.8)	30 (23.4)	26 (20.3)	21 (21.2)	25 (34.7)	100 (77.5)	10 (7.8)	4.62	1.44–14.8	0.006
Wild-Type	4 (3.1)	3 (2.3)	8 (6.3)	5 (3.9)	1 (1)	7 (9.7)	13 (10.1)	6 (4.7)			

CI, confidence interval.

## Discussion

We did not find any factor V Leiden mutation in any patients with ONFH or in any controls. Our results were consistent with those of Jun et al. [16], who found that none of their 369 Han Chinese patients with deep venous thrombosis and pulmonary embolism had factor V Leiden and concluded that “factor V Leiden and prothrombin G20210A mutations are very rare in the Chinese population”. Interruption of blood circulation has been suggested as the common pathogenesis pathway for the ONFH. The high incidence of patients in whom both hips are involved also suggests that it might be associated with genetic or constitutional traits [2], [20]. SNPs are the most common type of sequence variation and might be involved in common, polygenic diseases [21], [22]. Many SNPs have been related to hereditary thrombophilia or hypofibrinolysis and associated with a high prevalence rate in patients with ONFH [23]. The most frequently reported SNPs include factor V Leiden, prothrombin 20210A, plasminogen activator inhibitor-1, apolipoprotein, and the MTHFR gene [4]–[11]. However, these results were reported from the western countries that had different ethnic population as the Asian countries. In Asian populations, no significant differences in factor V Leiden and prothrombin 20210A have been reported between patients with ONFH and controls [12], [14], [15], [24]. The thrombophilic MTHFR C677T polymorphism was also contradictorily reported [12], [15]. Based on our findings and the overwhelming supportive evidence in the literature, we conclude that factor V Leiden is not responsible for the high prevalence of ONFH in the Chinese population.

Because the prevalence of ONFH in the Asian population is higher than in the Caucasian population, we hypothesized that Chinese patients with ONFH should also have a high frequency of coagulation abnormalities and coagulation gene SNPs than do healthy controls. We found that 87.6% of our patients with ONFH had one or more coagulation abnormalities related to fibrinogen, a fibrinogen degradation product, D-dimer, protein S, protein C, or anti-thrombin III. These findings were consistent with those reported [4], [6] in North America that around 74% of their patients had coagulation disorders. Our SNP microarray analysis found 6 distinct SNP loci in the factor V gene that were associated with increased risks of ONFH.

In the coagulation cascade, factor V promotes factor-X-catalyzed prothrombin activation and is inactivated by the activated protein C (APC)/protein S complex via proteolytic cleavage at the A2 domain. Factor V Leiden replaces arginine for glutamine (G-to-A) at position 506 because of a single-point mutation in the FV gene. This mutation alters the cleavage site at the A2 domain and is responsible for the APC-resistant thrombophilia phenotype [13], [25], [26]. The allelic frequency of factor V Leiden in the general Caucasian population is about 1-5.3% in contrast to 13.1–24% in patients with ONFH [5], [10], [11], [13]. We found that the genotype frequency of factor V Leiden (rs6025) was 0% in all study participants; the G-to-A genotype frequencies of rs6020 were 82.1% in patients with idiopathic ONFH, 88.9% in all patients with ONFH, and 88.6% in controls. Surprisingly, the minor allele frequency of rs6020 in the Caucasian population was 0% [13]. Although the exact molecular mechanism for APC resistance in carriers of the rs6020 polymorphism is still unclear, its association with coronary artery disease, thrombosis, and pre-eclampsia has been reported in the Asian population [27–29]. In our study, patients who had the rs6020 polymorphism also had a higher risk for coagulation abnormalities (odds ratio: 4.62). We hypothesize that the rs6020 polymorphism is the genetic trait that accounts for the higher prevalence of ONFH in the Chinese population the Caucasian population. Having the rs6020 polymorphism and being exposed to risk factors such as alcohol and steroids should lead to coagulation abnormalities and, subsequently, thromboembolisms in the femoral head. We also found 5 other SNP loci (rs9332595, rs9332647, rs3766110, rs10919186, and rs12040141) in the intron region of the factor V gene that were associated with an increased risk of ONFH. These SNPs might serve as a screening tool in patients who are at risk for developing osteonecrosis.

This study has limitations. First, the sample size, although larger than many of the genetic association studies on ONFH, is still relatively smaller than population-based genome-wide association studies. Second, this was not a prospective cohort study but a cross-sectional study with one control group. We do not know whether the controls with the rs6020 polymorphism exposed to risk factors in their later life will develop coagulation abnormalities or ONFH. Third, participants with known risk factors such as steroid exposure and alcohol abuse, and those with coagulation abnormalities on the screening test but without ONFH, were excluded from the study. Fourth, not all healthy controls had a radiographic examination of the pelvis. However, this study also has strengths. Comprehensive clinical information and laboratory tests for coagulation profiles were obtained. The microarray and SNP genotyping were done in a certified core facility with stringent quality control. (A parallel restriction fragment length polymorphism analysis was also done. Data not shown)

In conclusion, we confirmed that factor V Leiden was not associated with the increased risk in our population of developing ONFH. None of the patients with ONFH and none of the controls had factor V Leiden. We found 6 distinct loci of SNPs in the factor V gene that were associated with an increased risk of developing ONFH in Chinese patients. Most importantly, the rs6020 G-to-A polymorphism was highly prevalent in our Chinese population, but it is 0% in the Western population. The presence of the rs6020 G-to-A polymorphism predicted a high chance of coagulation abnormalities in patients with ONFH.

## Supporting Information

Table S1 Patient demographics, risk factors, and rs6020 polymorphism.
(DOC)

Table S2 rs Mutate and coagulopathy.
(DOC)
