# Supplementary material for: Single Nucleotide Polymorphisms Other than Factor V Leiden Are Associated with Coagulopathy and Osteonecrosis of the Femoral Head in Chinese Patients
Source: PLoS One. 2014 Aug 13;9(8):e104461. doi: 10.1371/journal.pone.0104461 (PMC4131902; doi:10.1371/journal.pone.0104461)
Supplement: Table S1 — Patient demographics, risk factors, and rs6020 polymorphism. (DOC) [file pone.0104461.s001.doc]

TableS1. Patient demographics, risk factors, and rs6020 polymorphism

sex side risk age RS6020

3 47.01.25 1 b a 46 AG

8 61.06.09 1 b a 33 AG

9 54.05.02 1 b a 40 AG

10 43.07.29 2 b st 51 AG

11 60.10.20 1 b a 34 AG

12 40.04.10 2 b i 49 AA

13 38.01.05 1 r a 57 AG

15 31.07.05 1 b a 63 AG

16 41.05.01 1 l a 53 AG

17 45.12.31 1 l i 49 AA

19 44.10.20 1 b a 49 AG

23 40.10.15 2 b i 54 AA

25 45.10.04 1 r a 49 AA

26 51.11.19 1 r a 43 AG

29 53.10.31 1 b st 41 AA

31 42.04.26 1 b a 52 AG

34 55.08.31. 1 r a 38 AA

35 47.10.30 1 l a 47 AG

38 45.03.21 1 b a 49 AA

41 37.03.22 1 b a 57 AA

45 54.01.21 1 b a 38 AG

46 55.01.25 1 b a 38 AA

47 47.08.10 1 b a 46 AG

50 55.01.01 1 b a 38 AG

53 45.06.24 2 r a 49 AG

54 36.06.22 1 b a 57 AG

55 39.08.15 1 b a 53 AA

56 58.10.04 1 b a 36 AA

58 65.03.11 1 b a 28 AG

61 30.02.13 1 b a 64 AA

62 43.07.08 2 b i 51 AG

63 49.12.03 2 l i 45 AA

64 63.10.03 1 l a 32 AG

65 52.01.22 1 l a 41 AG

66 50.08.12 1 r a 43 GG

67 43.08.20 1 r a 50 AG

68 41.09.02 1 b a 54 AA

70 47.01.21 1 b a 46 AG

71 61.09.10 1 r a 34 AG

72 36.12.12 1 b a 55 AG

73 71.10.05 2 r st 24 AG

74 51.08.10 1 b a 42 AG

77 44.10.10 1 r a 49 GG

81 55.01.22 1 r a 38 AA

85 59.07.10 1 b a 35 AG

86 35.08.18 2 r i 57 AG

87 30.05.06 1 b a 62 AA

88 42.03.11 1 b a 52 AA

89 38.09.07 1 b a 56 AG

92 49.11.07 2 b i 45 AA

93 55.01.25 1 b a 38 GG

94 44.02.06 1 l i 51 AG

95 58.12.07 1 r a 37 AG

96 36.11.14 1 b a 59 AG

97 71.05.06 2 b i 24 AG

98 47.04.10 1 b i 48 AG

99 66.04.18 2 b st 29 AG

100 50.05.20 1 r a 45 AG

101 56.04.10 1 b a 39 AA

102 45.01.12 1 b a 50 AG

103 67.09.11 2 b i 28 AA

104 50.04.02 1 b a 44 AG

105 49.11.04 1 b a 44 AG

106 15.05.31 1 r i 80 GG

107 62.02.12 1 b a 29 AG

108 51.04.20 1 b st 44 AG

109 38.09.05 2 l i 57 GG

111 45.01.10 1 l a 50 AG

113 43.07.02 2 l i 52 AG

115 61.02.04 2 b st 34 AA

116 63.11.02 1 b i 32 GG

117 39.05.10 1 b a 56 AA

118 60.12.15 1 r i 35 AG

119 47.05.02 2 b i 47 0

120 42.04.20 1 b a 53 AG

122 47.09.03 1 b a 46 AG

123 71.08.17 1 b i 24 AG

126 65.09.13 1 b a 30 AA

127 49.11.20 1 l a 46 AG

132 55.10.15 1 l i 40 GG

133 40.12.01 2 l i 56 GG

134 48.05.17 1 b a 47 AG

135 56.07.06 1 b a 39 AG

136 41.05.02 1 b a 55 AA

137 40.01.03 1 r a 56 GG

138 51.12.28 1 b a 45 AG

140 40.08.18 1 b a 56 GG

141 62.02.03 1 r a 34 AG

142 56.11.06 1 b a 37 AA

143 59.02.18 1 l a 37 AA

144 77.05.21 2 b st 19 GG

145 65.08.07 1 b i 28 GG

146 69.11.07 1 r i 27 AA

148 62.05.31 2 r i 34 AA

149 45.08.29 1 b a 50 AG

150 60.02.12 1 r i 36 AG

151 33.09.01 2 b i 61 AA

152 50.12.04 1 b a 48 AG

153 52.09.10 2 b i 40 AA

154 56.12.20 2 b i 40 AG

155 28.07.08 1 b a 65 AA

156 34.07.08 2 b st 62 AG

158 51.07.27 1 b a 43 AG

159 73.07.29 2 b st 20 AG

160 44.05.09 1 b a 48 AG

161 36.12.01 2 b i 61 GG

162 45.12.27 1 b st 46 AA

163 38.03.08 1 b a 53 AA

164 55.02.16 1 b a 36 GG

165 38.11.20 1 r a 52 AA

166 29.10.27 2 r i 60 AA

167 36.03.20 1 r i 56 AG

168 16.03.03 2 b st 78 AA

170 60.12.24 1 b a 37 AA

171 63.10.12 1 b i 34 AG

173 39.06.25 1 b a 54 AG

174 65.06.01 1 b a 33 AA

175 65.03.05 2 b i 33 AA

176 67.01.10 1 b i 31 AG

177 69.06.01 2 b st 24 GG

178 61.01.27 2 r i 37 AA

179 52.08.23 1 r a 46 AA

180 59.11.18 1 r i 39 AA

182 64.08.01 1 r i 30 AA

183 45.06.06 1 b a 47 GG

184 37.11.16 1 r a 60 AA

185 39.12.17 1 b st 55 AG

186 72.10.12 2 b i 26 AG

187 55.08.12 1 b a 38 AA

188 59.02.18 1 r a 32 AA

189 58.01.22 1 r a 36 AA

190 52.09.29 2 b i 46 AA

191 53.09.20 2 b i 45 AG

192 46.01.10 1 b a 48 AG

AVN001 44.06.18 1 b a 57 AG

AVN002 51.5.21 2 r i 50 AG

AVN003 52.03.10 1 b a 49 AA

AVN004 1 b a 57 AG

AVN005 54.03.05 1 b a 47 AA

AVN007 60.01.11 1 b a 41 AA

AVN008 24.08.12 1 b a 73 AA

AVN009 48.12.08 1 b a 52 AA

AVN010 27.09.21 2 r st 74 AA

AVN011 51.05.28 2 r st 50 AG

AVN013 65.05.27 1 b st 36 AA

AVN014 63.04.18 2 b a 37 AG
