# Supplementary material for: Single Nucleotide Polymorphisms Other than Factor V Leiden Are Associated with Coagulopathy and Osteonecrosis of the Femoral Head in Chinese Patients
Source: PLoS One. 2014 Aug 13;9(8):e104461. doi: 10.1371/journal.pone.0104461 (PMC4131902; doi:10.1371/journal.pone.0104461)
Supplement: Table S2 — rs Mutate and coagulopathy. (DOC) [file pone.0104461.s002.doc]

Table S2. RS mutate and coagulopathy

|  | 4524 | 974793 | 3766110 | 3820060 | 4656685 | 6662593 | 9332595 | 9332600 | 9332619 | 9332627 | 9332647 | 10919186 | 12040141 | V11216153 | V12121543 | V1801133 | V2227631 | V6020 | Vrs2292566 |
| --- | --- | --- | --- | --- | --- | --- | --- | --- | --- | --- | --- | --- | --- | --- | --- | --- | --- | --- | --- |
| group | (-) | (+) | (+) | (+) | (+) | (+) | (-) | (-) | (-) | (-) | (-) | (+) | (+) | (+) | (+) | (-) | (+) | (-) | (+) |
| AVN | GG | TT | AC | GG | TT | AA | CG | AA | TT | TT | AA | CC | AA | GG | CC | TC | AA | AG | AG |
| AVN | AG | CT | AC | GT | CT | AG | CG | AG | CT | CT | AG | CC | AG | GG | CC | TT | GG | AG | AA |
| AVN | AG | CT | AC | GT | CT | 0 | 0 | 0 | CT | CT | AG | CC | AG | GT | CC | 0 | GG | AG | AA |
| AVN | AA | CC | AA | TT | CC | GG | CC | GG | CC | CC | AG | CG | AG | GT | AA | CC | AG | AG | GG |
| AVN | AG | CT | AC | GT | CT | AG | CG | AG | CT | CT | AG | CC | AG | GG | CC | CC | GG | AG | GG |
| AVN | AA | CC | AA | TT | CC | GG | CC | GG | CC | CC | GG | CG | GG | GG | CA | CC | AG | AA | GG |
| AVN | AG | CT | AC | GT | CT | AG | CG | AG | CT | CT | AG | CC | AG | GT | CC | TC | AA | AG | GG |
| AVN | AG | CT | AC | GT | CT | AG | CG | AG | CT | CT | AG | CC | AG | GG | CA | TC | GG | AG | AG |
| AVN | AA | CC | AA | TT | CC | GG | CC | GG | CC | CC | AG | CG | AG | GG | CC | TC | AA | AG | AG |
| AVN | AA | CC | AA | TT | CC | GG | CC | GG | CC | CC | GG | CG | GG | TT | CC | CC | AG | AA | AG |
| AVN | AA | CC | AA | TT | CC | GG | CC | GG | CC | CC | AG | CG | AG | 0 | 0 | CC | AG | AG | GG |
| AVN | AA | CC | AA | TT | CC | GG | CC | GG | CC | CC | GG | CG | GG | GG | AA | CC | GG | AA | AG |
| AVN | AA | CC | AA | TT | CC | GG | CC | GG | CC | CC | GG | CG | GG | TT | CA | CC | GG | AA | GG |
| AVN | AG | CT | AC | GT | CT | AG | CG | AG | CT | CT | AG | CC | AG | GG | CA | CC | AG | AG | AG |
| AVN | AA | CC | AA | TT | CC | GG | CC | GG | CC | CC | GG | CG | GG | GT | CA | TC | GG | AA | GG |
| AVN | AG | CT | AC | GT | CT | AG | CG | AG | CT | CT | AG | CC | AG | GT | CA | CC | AG | AG | GG |
| AVN | AA | CC | AA | TT | CC | GG | CC | GG | CC | CC | GG | CG | GG | GT | CA | TC | GG | AA | AG |
| AVN | AA | CC | AA | TT | CC | GG | CC | GG | CC | CC | AG | CG | AG | GT | AA | CC | AA | AG | AG |
| AVN | AA | CC | AA | TT | CC | GG | CC | GG | CC | CC | GG | CG | GG | GT | CA | CC | AG | AA | GG |
| AVN | AA | CC | AA | TT | CC | GG | CC | GG | CC | CC | GG | CG | GG | GT | CA | TC | AG | AA | AA |
| AVN | AG | CT | AC | GT | CT | AG | CG | AG | CT | CT | AG | CC | AG | GT | CC | TC | AG | AG | AG |
| AVN | AA | CC | AA | TT | CC | GG | CC | GG | CC | CC | GG | CG | GG | GT | CC | CC | AG | AA | AG |
| AVN | AG | CT | AC | GT | CT | AG | CG | AG | CT | CT | AG | CC | AG | TT | CC | TC | GG | AG | GG |
| AVN | AA | CC | AA | TT | CC | GG | CC | GG | CC | CC | AG | CG | AG | GG | AA | CC | AG | AG | GG |
| AVN | AA | CC | AA | TT | CC | GG | CC | GG | CC | CC | GG | CG | GG | GG | CA | CC | AG | AG | GG |
| AVN | AG | CT | AC | GT | CT | AG | CG | AG | CT | CT | AG | CC | AG | GT | CC | TC | GG | AG | GG |
| AVN | AA | CC | AA | TT | CC | GG | CC | GG | CC | CC | GG | CG | GG | GT | CA | CC | AG | AA | GG |
| AVN | AA | CC | AA | TT | CC | GG | CC | GG | CC | CC | GG | CG | GG | GG | CA | TC | AG | AA | GG |
| AVN | AA | CC | AA | TT | CC | GG | CC | GG | CC | CC | AG | CG | AG | GG | CA | CC | AG | AG | GG |
| AVN | AA | CC | AA | TT | CC | GG | CC | GG | CC | CC | GG | CG | GG | GG | CA | CC | AG | AA | GG |
| AVN | AA | CC | AA | TT | CC | GG | CC | GG | CC | CC | AG | CG | AG | TT | CA | CC | AG | AG | GG |
| AVN | AA | CC | AA | TT | CC | GG | CC | GG | CC | CC | GG | CG | GG | GT | CA | CC | GG | AA | GG |
| AVN | AA | CC | AA | TT | CC | GG | CC | GG | CC | CC | AG | CG | AG | GG | CC | CC | GG | AG | GG |
| AVN | AG | CT | AC | GT | CT | AG | CG | AG | CT | CT | AG | CC | AG | TT | AA | CC | GG | AG | GG |
| AVN | AG | CT | AC | GT | CT | AG | CG | AG | CT | CT | AA | CC | AA | GG | CC | CC | AG | GG | AG |
| AVN | AA | CC | AA | TT | CC | GG | CC | GG | CC | CC | AG | CG | AG | GT | CC | CC | AG | AG | AG |
| AVN | AA | CC | AA | TT | CC | GG | CC | GG | CC | CC | GG | CG | GG | GG | CC | TT | AA | AA | GG |
| AVN | AG | CT | AC | GT | CT | AG | CG | AG | CT | CT | AG | CC | AG | GT | AA | CC | GG | AG | GG |
| AVN | AA | CC | AA | TT | CC | GG | CC | GG | CC | CC | AG | CG | AG | GT | CC | TT | AG | AG | GG |
| AVN | AG | CT | AC | GT | CT | AG | CG | AG | CT | CT | AG | CC | AG | GT | CC | TC | GG | AG | GG |
| AVN | AG | CT | AC | GT | CT | AG | CG | AG | CT | CT | AG | CC | AG | GG | CA | CC | GG | AG | GG |
| AVN | AA | CC | AA | TT | CC | GG | CC | GG | CC | CC | AG | CG | AG | GT | CC | TC | GG | AG | GG |
| AVN | AA | CC | AA | TT | CC | GG | CC | GG | CC | CC | AA | CG | AA | GT | CA | TC | GG | GG | AG |
| AVN | AA | CC | AA | TT | CC | GG | CC | GG | CC | CC | GG | CG | GG | GG | CC | CC | AG | AA | AG |
| AVN | AA | CC | AA | TT | CC | GG | CC | GG | CC | CC | AG | CG | AG | TT | CC | TC | AG | AG | GG |
| AVN | AG | CT | AC | GT | CT | AG | CG | AG | CT | CT | AG | CC | AG | GG | CC | TC | AG | AG | GG |
| AVN | AA | CC | AA | TT | CC | GG | CC | GG | CC | CC | GG | CG | GG | GT | CC | CC | AA | AA | AG |
| AVN | AA | CC | AA | TT | CC | GG | CC | GG | CC | CC | GG | CG | GG | GT | CC | TC | AG | AA | AG |
| AVN | AG | CT | AC | GT | CT | AG | CG | AG | CT | CT | AG | CC | AG | GG | CA | CC | GG | AG | GG |
| AVN | AA | CC | AA | TT | CC | GG | CC | GG | CC | CC | GG | CG | GG | GG | CC | TC | AA | AA | GG |
| AVN | AA | CC | AC | TT | CC | GG | CG | GG | CC | CC | AG | CC | AG | GG | CC | TC | AA | GG | AG |
| AVN | AA | CC | AA | TT | CC | GG | CC | GG | CC | CC | AG | CG | AG | GT | CC | CC | GG | AG | GG |
| AVN | AG | CT | AC | GT | CT | AG | CG | AG | CT | CT | AG | CC | AG | GG | AA | CC | AG | AG | GG |
| AVN | AG | CT | AC | GT | CT | AG | CG | AG | CT | CT | AG | CC | AG | GT | CC | CC | AG | AG | GG |
| AVN | AG | CT | AA | GT | CT | AG | CC | AG | CT | CT | AA | CG | AA | GG | CC | CC | AA | AG | AG |
| AVN | GG | TT | AC | GG | TT | AA | CG | AA | TT | TT | AA | CC | AA | GG | CA | CC | AA | AG | AA |
| AVN | AA | CC | AA | GT | CC | GG | CC | GG | CC | CC | AG | CG | AG | GG | CC | TT | AG | AG | AG |
| AVN | AG | CT | AC | GT | CT | AG | CG | AG | CT | CT | AG | CC | AG | GT | AA | CC | GG | AG | GG |
| AVN | AA | CC | AA | TT | CC | GG | CC | GG | CC | CC | GG | CG | GG | GT | CC | CC | AG | AA | AG |
| AVN | AA | CC | AA | TT | CC | GG | CC | GG | CC | CC | AG | CG | AG | GG | CC | TC | AG | AG | GG |
| AVN | AA | CC | AA | TT | CC | GG | CC | GG | CC | CC | GG | CG | GG | GG | CA | CC | AG | AA | AA |
| AVN | AA | CC | AC | TT | CC | GG | CG | GG | CC | CC | GG | CC | GG | TT | CC | TC | AG | AG | AA |
| AVN | AG | CT | AC | GT | CT | AG | CG | AG | CT | CT | AG | CC | AG | GT | CA | CC | AA | AG | GG |
| AVN | GG | TT | CC | GG | TT | AA | GG | AA | TT | TT | AA | CC | AA | GT | CC | CC | AG | GG | GG |
| AVN | AG | CT | AC | GT | CT | AG | CG | AG | CT | CT | AG | CC | AG | GT | CC | CC | GG | AG | AG |
| AVN | AA | CC | AA | TT | CC | GG | CC | GG | CC | CC | AG | CG | AG | GG | CC | CC | AA | AG | GG |
| AVN | AG | CT | AC | GT | CT | AG | CG | AG | CT | CT | AA | CC | AA | GT | CC | CC | AG | GG | GG |
| AVN | AG | CT | AC | GT | CT | AG | CG | AG | CT | CT | AG | CC | AG | GG | CC | CC | GG | AG | GG |
| AVN | AA | CC | AA | GT | CC | GG | CC | GG | CC | CC | AG | CG | AG | GG | CA | CC | GG | AG | GG |
| AVN | AA | CC | AA | TT | CC | GG | CC | GG | CC | CC | GG | CG | GG | GG | CA | CC | AG | AA | AG |
| AVN | AA | CC | AC | GT | CC | GG | CG | GG | CC | CC | AG | CC | AG | GT | CC | CC | AA | GG | AG |
| AVN | AA | CT | AA | GT | CT | GG | CC | GG | CT | CT | AG | CG | AG | GG | CC | CC | AG | AA | AG |
| AVN | AG | CT | AC | GT | CT | AG | CG | AG | CT | CT | AG | CC | AG | GT | AA | CC | GG | AG | GG |
| AVN | 0 | CT | AC | GT | CT | AG | CG | AG | CT | CT | 0 | CC | AG | GG | CC | CC | AA | 0 | AG |
| AVN | AA | CC | AA | TT | CC | GG | CC | GG | CC | CC | GG | CG | AG | GG | CA | CC | GG | AG | AG |
| AVN | AA | CC | AC | TT | CC | GG | CG | GG | CC | CC | GG | CC | GG | GG | CC | TT | GG | AG | GG |
| AVN | AG | CT | AC | GT | CT | AG | CG | AG | CT | CT | AG | CC | AG | GG | CC | TC | AG | AG | AG |
| AVN | AA | CC | AA | TT | CC | GG | CC | GG | CC | CC | GG | CG | GG | GT | CC | TC | AG | AA | AG |
| AVN | AA | CC | AA | TT | CC | GG | CC | GG | CC | CC | AG | CG | AG | GG | CA | CC | AG | AG | GG |
| AVN | AG | CT | AC | GT | CT | AG | CG | AG | CT | CT | AA | CC | AA | GT | CC | TC | AA | GG | GG |
| AVN | GG | TT | CC | GG | TT | AA | GG | AA | TT | TT | AA | CC | AA | GT | CA | TC | AG | GG | AG |
| AVN | AG | CT | AA | GT | CT | AG | CC | AG | CT | CT | AA | CG | AA | GT | CC | TC | AG | AG | AG |
| AVN | AA | CC | AC | TT | CC | GG | CG | GG | CC | CC | GG | CC | GG | GT | CC | TC | AG | AG | GG |
| AVN | AA | CC | AA | TT | CC | GG | CC | GG | CC | CC | GG | CG | GG | GT | CC | TC | GG | AA | GG |
| AVN | AA | CC | AA | TT | CC | GG | CC | GG | CC | CC | AA | CG | AA | GT | CC | TC | AG | GG | GG |
| AVN | AG | CT | AC | GT | CT | AG | CG | AG | CT | CT | AG | CC | AG | GT | CC | TC | GG | AG | AG |
| AVN | AG | CT | AC | GT | CT | AG | CG | AG | CT | CT | AA | CC | AA | TT | CA | CC | GG | GG | AG |
| AVN | AG | CT | AC | GT | CT | AG | CG | AG | CT | CT | AG | CC | AG | GG | CA | CC | AG | AG | AG |
| AVN | AA | CC | AA | TT | CC | GG | CC | GG | CC | CC | GG | CG | GG | 0 | CC | 0 | GG | AA | 0 |
| AVN | AA | CC | AA | TT | CC | GG | CC | GG | CC | CC | GG | CG | GG | TT | CC | CC | GG | AA | AG |
| AVN | GG | CT | CC | GT | CT | AA | GG | AA | TT | TT | AG | CC | AA | GG | CC | TC | AG | GG | AG |
| AVN | GG | TT | CC | GG | TT | AA | GG | AA | TT | TT | AA | CC | AA | GG | CC | CC | GG | GG | GG |
| AVN | AA | CC | AA | TT | CC | GG | CC | GG | CC | CC | GG | CG | GG | GG | CC | TC | GG | AA | GG |
| AVN | AA | CC | AA | TT | CC | GG | CC | GG | CC | CC | GG | CG | GG | GT | CC | TC | AA | AA | AG |
| AVN | AA | CC | AA | TT | CC | GG | CC | GG | CC | CC | AG | CG | AG | GT | CA | CC | AG | AG | AA |
| AVN | AG | CT | AA | GT | CT | AG | CC | AG | CT | CT | AA | CG | AA | GT | CC | TC | AA | AG | GG |
| AVN | AA | CC | AA | TT | CC | GG | CC | GG | CC | CC | GG | CG | GG | GG | 0 | TC | AA | AA | AG |
| AVN | AG | CT | AC | GT | CT | AG | CG | AG | CT | CT | AG | CC | AG | GG | CA | CC | AG | AG | GG |
| AVN | AA | CC | AA | TT | CC | GG | CC | GG | CC | CC | GG | CG | GG | GT | CC | TC | AA | AA | GG |
| AVN | AG | CT | AC | GT | CT | AG | CG | AG | CT | CT | AG | CC | AG | GT | CA | CC | AA | AG | AA |
| AVN | AA | CC | AA | TT | CC | GG | CC | GG | CC | CC | GG | CG | GG | GG | CC | TC | AG | AA | GG |
| AVN | AA | CC | AA | TT | CC | GG | CC | GG | CC | CC | AG | CG | AG | GT | CC | CC | GG | AG | AA |
| AVN | AG | CT | AC | GT | CT | AG | CG | AG | CT | CT | AG | CC | AG | GG | CA | CC | GG | AG | AG |
| AVN | AA | CC | AA | TT | CC | GG | CC | GG | CC | CC | AG | CG | AG | GG | CA | TC | GG | AG | AG |
| AVN | AA | CC | AA | TT | CC | GG | CC | GG | CC | CC | AG | 0 | AG | GG | CA | TC | GG | AG | AG |
| AVN | AG | CT | AC | GT | CT | AG | CG | AG | CT | CT | AG | CC | AG | GT | CC | CC | AA | GG | GG |
| AVN | AA | CC | AA | TT | CC | GG | CC | GG | CC | CC | GG | CC | GG | GG | CC | CC | AG | AA | GG |
| AVN | AA | CC | AA | TT | CC | GG | CC | GG | CC | CC | GG | CG | GG | TT | CA | TC | AG | AA | GG |
| AVN | AG | CT | AC | GT | CT | AG | CG | AG | CT | CT | AA | CC | AA | GG | CA | CC | AG | GG | GG |
| AVN | AA | CC | AA | TT | CC | GG | CC | GG | CC | CC | GG | CG | GG | GG | CC | CC | GG | AA | AG |
| AVN | AA | CC | AA | TT | CC | GG | CC | GG | CC | CC | GG | CG | GG | GT | CC | TC | GG | AA | GG |
| AVN | AG | CT | AC | GT | CT | AG | CG | AG | CT | CT | AG | CC | AG | TT | CC | TC | AG | AG | AG |
| AVN | AA | CC | AA | TT | CC | GG | CC | GG | CC | CC | GG | CG | GG | GT | CA | CC | GG | AA | AG |
| AVN | AA | CC | AA | TT | CC | GG | CC | GG | CC | CC | GG | CG | GG | GG | CC | CC | AG | AA | AG |
| AVN | 0 | 0 | 0 | 0 | 0 | 0 | 0 | 0 | 0 | CC | AG | CG | AG | 0 | CC | 0 | AG | AG | AG |
| AVN | AG | CT | AC | GT | CT | AG | CG | AG | CT | CT | AG | CC | AG | GG | CA | CC | AA | AG | AA |
| AVN | AA | CC | AA | TT | CC | GG | CC | GG | CC | CC | GG | CG | GG | TT | CC | CC | AG | AA | AG |
| AVN | AA | CC | AA | TT | CC | GG | CC | GG | CC | CC | GG | CG | GG | GG | CA | CC | AG | AA | GG |
| AVN | AA | CC | AA | TT | CC | GG | CC | GG | CC | CC | AG | CG | AG | GT | CC | TC | AG | AG | GG |
| AVN | GG | CT | 0 | GG | CT | AG | CG | 0 | 0 | CT | AA | CC | AA | GG | 0 | CC | GG | GG | GG |
| AVN | AA | CC | AA | TT | CC | GG | CC | GG | CC | CC | GG | CG | GG | GG | CA | TC | GG | AA | AG |
| AVN | AA | CC | AA | TT | CC | GG | CC | GG | CC | CC | GG | CG | GG | GG | CC | TC | 0 | AA | GG |
| AVN | AA | CT | AA | GT | CT | GG | CC | GG | CT | CT | AG | CG | AG | GT | CA | CC | GG | AA | AG |
| AVN | AA | CC | AA | TT | CC | GG | CC | GG | CC | CC | GG | CG | GG | GT | CC | TC | AG | AA | AG |
| AVN | AG | CT | AC | GT | CT | AG | CG | AG | CT | CT | AA | CC | AA | GT | CC | CC | AG | GG | AG |
| AVN | AA | CC | AA | TT | CC | GG | CC | GG | CC | CC | GG | CG | GG | 0 | CC | TC | AG | AA | 0 |
| AVN | AG | CT | AC | GT | CT | AG | CG | AG | CT | CT | AG | CC | AG | GG | CA | CC | GG | AG | GG |
| AVN | AG | CT | AC | GT | CT | AG | CG | AG | CT | CT | AG | CC | AG | GT | CC | CC | AA | AG | AG |
| AVN | AA | CC | AA | TT | CC | GG | CC | GG | CC | CC | GG | CG | GG | GG | CC | CC | AA | AA | GG |
| AVN | AA | CC | AA | TT | CC | GG | CC | GG | CC | CC | GG | CG | GG | GT | CC | TC | GG | AA | AG |
| AVN | AA | CC | AA | TT | CC | GG | CC | GG | CC | CC | GG | CG | GG | GG | CA | CC | AA | AA | AG |
| AVN | AA | CC | AA | TT | CC | GG | CC | GG | CC | CC | GG | CG | GG | GT | CA | CC | AG | AA | GG |
| AVN | AA | CT | AA | GT | CT | GG | CC | GG | CT | CT | AG | CG | AG | GG | CA | CC | AG | AG | AG |
| AVN | AG | CT | AC | GT | CT | AG | CG | AG | CT | CT | AG | CC | AG | GG | CC | CC | AG | AG | GG |
| AVN | AA | CC | AA | GT | CC | GG | CC | GG | CC | CC | AG | CG | AG | GT | CC | TC | AG | AG | GG |
| AVN | AA | CC | AC | TT | CC | GG | CG | GG | CC | CC | GG | CC | GG | GT | CC | CC | AA | AG | AG |
| AVN | AA | CC | AA | TT | CC | GG | CC | GG | CC | CC | GG | CG | GG | GG | CC | CC | GG | AA | AG |
| AVN | AA | CC | AA | TT | CC | GG | CC | GG | CC | CC | AG | CG | AG | GG | CA | CC | AG | AG | AG |
| AVN | AA | CC | AA | TT | CC | GG | CC | GG | CC | CC | GG | CG | GG | GT | CA | CC | GG | AA | AG |
| AVN | AA | CC | AA | TT | CC | GG | CC | GG | CC | CC | GG | CG | GG | GT | CC | CC | AA | AA | GG |
| AVN | AA | CC | AA | TT | CC | GG | CC | GG | CC | CC | GG | CG | GG | GT | CC | TC | AG | AA | AG |
| AVN | AA | CC | AA | TT | CC | GG | CC | GG | CC | CC | GG | CG | GG | GT | CA | CC | AA | AA | GG |
| AVN | AA | CC | AA | TT | CC | GG | CC | GG | CC | CC | GG | CG | GG | GT | CC | CC | GG | AA | AA |
| AVN | AG | CT | AC | GT | CT | AG | CG | AG | CT | CT | AG | CC | AG | GG | CA | CC | AA | AG | AG |
| AVN | AA | CC | AA | TT | CC | GG | CC | GG | CC | CC | GG | CG | GG | GT | CC | TC | GG | AA | AG |
| AVN | AG | CT | AC | GT | CT | AG | CG | AG | CT | CT | AG | CC | AG | TT | CC | TC | GG | AG | GG |
| CTRL | AA | CC | AA | TT | CC | GG | CC | GG | CC | CC | AG | CG | AG | GG | CC | TC | AA | AG | AG |
| CTRL | AA | CC | AA | TT | CC | GG | CC | GG | CC | CC | GG | CG | GG | GT | CA | CC | AG | AA | AA |
| CTRL | 0 | CT | AC | GT | CT | AG | CG | AG | 0 | CT | AG | CC | AG | TT | CA | TC | 0 | AG | GG |
| CTRL | 0 | 0 | 0 | 0 | 0 | 0 | 0 | 0 | 0 | CC | 0 | CC | 0 | 0 | CA | 0 | 0 | 0 | GG |
| CTRL | AA | CC | AA | TT | CC | GG | CC | GG | CC | CC | AG | CG | AG | GT | CC | CC | AG | AG | GG |
| CTRL | AA | CC | AA | TT | CC | GG | CC | GG | CC | CC | GG | CG | GG | GG | CC | TT | AG | AA | AG |
| CTRL | AA | CC | AA | TT | CC | GG | CC | GG | CC | CC | GG | CG | GG | GT | CC | TT | AG | AA | AG |
| CTRL | AG | CT | AC | 0 | 0 | AG | CG | AG | CT | CT | AG | CC | AG | GT | CC | TC | AG | AG | GG |
| CTRL | AG | CT | AC | GT | CT | AG | CG | AG | CT | CT | AA | CC | AA | GT | CC | TC | AG | GG | AG |
| CTRL | AA | CC | AA | TT | CC | GG | CC | GG | CC | CC | GG | CG | GG | GT | CC | TC | GG | AA | AG |
| CTRL | AA | CC | AA | TT | CC | GG | CC | GG | CC | CC | GG | CG | GG | GT | CC | TC | GG | AA | GG |
| CTRL | AG | CT | AC | GT | CT | AG | CG | AG | CT | CT | AG | CC | AG | GT | CC | TC | AG | AG | AG |
| CTRL | AA | CC | AA | TT | CC | GG | CC | GG | CC | CC | AG | CG | AG | GT | CA | CC | AG | AG | AG |
| CTRL | AA | CC | AA | TT | CC | GG | CC | GG | CC | CC | GG | CG | GG | GG | CA | CC | AG | AA | AA |
| CTRL | AA | CC | AA | TT | CC | GG | CC | GG | CC | CC | GG | CG | GG | GT | CC | CC | AG | AA | GG |
| CTRL | AG | CT | AC | GG | CT | AG | CG | AG | CT | CT | AA | CC | AA | GG | CC | TC | GG | GG | GG |
| CTRL | AG | CT | AC | GT | CT | AG | CG | AG | CT | CT | AG | 0 | AG | GT | CC | CC | GG | AG | AG |
| CTRL | AA | CC | AA | TT | CC | GG | CC | GG | CC | CC | GG | CG | GG | GG | CC | CC | AG | AA | GG |
| CTRL | AG | CT | AC | GT | CT | AG | CG | AG | CT | CT | AG | CC | AG | GT | CC | CC | AG | 0 | GG |
| CTRL | AG | CT | CC | GT | CT | AG | GG | AG | CT | CT | AG | CC | AG | GT | CA | CC | GG | GG | AA |
| CTRL | AG | CT | AC | GT | CT | AG | CG | AG | CT | CT | AG | CC | AG | GT | CC | CC | AG | AG | AG |
| CTRL | AA | CC | AA | TT | CC | GG | CC | GG | CC | CC | AG | CG | AG | GG | CC | TT | GG | AG | GG |
| CTRL | AA | CC | AA | TT | CC | GG | CC | GG | CC | CC | GG | CG | GG | GT | CC | TC | AG | AA | GG |
| CTRL | AA | CC | AA | TT | CC | GG | CC | GG | CC | CC | GG | CG | GG | GT | CC | TC | GG | AA | GG |
| CTRL | AA | CC | AC | TT | CC | GG | CG | GG | CC | CC | GG | CC | GG | 0 | CC | TC | AG | AG | AG |
| CTRL | GG | TT | CC | GG | TT | AA | GG | AA | TT | TT | AA | CC | AA | GT | CC | TC | AA | GG | GG |
| CTRL | AG | CT | AC | GT | CT | AG | CG | AG | CT | CT | AA | CC | AA | GT | CC | TC | AG | GG | GG |
| CTRL | AA | CC | AA | TT | CC | GG | CC | GG | CC | CC | AG | CG | AG | GT | CA | CC | AG | AG | GG |
| CTRL | AA | CC | AA | TT | CC | GG | CC | GG | CC | CC | GG | CG | GG | GG | CC | TC | AA | AA | AG |
| CTRL | AG | CT | AC | GT | CT | AG | CG | AG | CT | CT | AG | CC | AG | GT | CA | CC | GG | AG | GG |
| CTRL | AG | CT | AC | GT | CT | AG | CG | AG | CT | CT | AA | CC | AA | GT | CC | TC | GG | GG | AG |
| CTRL | AA | CC | AA | TT | CC | GG | CC | GG | CC | CC | GG | CG | GG | GG | CC | TC | AG | AA | GG |
| CTRL | AA | CC | AA | TT | CC | GG | CC | GG | CC | CC | GG | CG | GG | GG | CC | TC | AA | AA | GG |
| CTRL | AG | CT | AC | GT | CT | AG | CG | AG | CT | CT | AG | CC | AG | GT | CC | TC | AG | AG | GG |
| CTRL | AA | CC | AA | TT | CC | GG | CC | GG | CC | CC | GG | CG | GG | GG | CC | TC | GG | AA | AG |
| CTRL | AA | CC | AA | TT | CC | GG | CC | GG | CC | CC | GG | CG | GG | TT | CC | TC | GG | AA | GG |
| CTRL | AA | CC | AA | TT | CC | GG | CC | GG | CC | CC | GG | CG | GG | GG | CC | CC | AA | AA | GG |
| CTRL | AA | CC | AA | TT | CC | GG | CC | GG | CC | CC | AG | CG | AG | GT | AA | CC | AG | AG | AG |
| CTRL | AA | CC | AA | TT | CC | GG | CC | GG | CC | CC | GG | CG | GG | GG | CC | TC | GG | AA | GG |
| CTRL | AA | CC | AA | TT | CC | GG | CC | GG | CC | CC | GG | CG | GG | GT | 0 | CC | AG | AA | 0 |
| CTRL | AA | CC | AA | TT | CC | GG | CC | GG | CC | CC | GG | CG | GG | GT | CA | CC | AG | AA | GG |
| CTRL | AA | CC | AA | TT | CC | GG | GG | GG | CC | CC | GG | CC | GG | GG |  |  | AG | AA |  |
| CTRL | AA | CC | AA | TT | CC | GG | GG | GG | CC | CC | GG | CC | GG | GG |  |  | GG | AA |  |
| CTRL | AA | CC | AA | TT | CC | GG | GG | GG | CC | CC | GG | CC | GG | GT |  |  | GG | AA |  |
| CTRL | AA | CC | AA | TT | CC | GG | GG | GG | CC | CC | GG | CC | GG | GT |  |  | GG | AA |  |
| CTRL | AG | CT | AA | GT | CT | AG | GG | AG | CT | CT | AG | CC | AG | GT |  |  | GG | AA |  |
| CTRL | AA | CC | AA | TT | CC | GG | GG | GG | CC | CC | GG | CC | GG | GT |  |  | GG | AA |  |
| CTRL | AA | CC | AA | TT | CC | GG | GG | GG | CC | CC | GG | CC | GG | GG |  |  | GG | AA |  |
| CTRL | AG | CT | AA | GT | CT | AG | GG | AG | CT | CT | AG | CC | AG | GG |  |  | GG | AA |  |
| CTRL | AG | CT | AA | GT | CT | AG | GG | AG | CT | CT | AG | CC | AG | GT |  |  | GG | AA |  |
| CTRL | AA | CC | AA | TT | CC | GG | GG | GG | CC | CC | GG | CC | GG | GT |  |  | GG | AA |  |
| CTRL | AA | CC | AA | TT | CC | GG | GG | GG | CC | CC | GG | CC | GG | GG |  |  | GG | AA |  |
| CTRL | AA | CC | AA | GT | CC | GG | GG | GG | CC | CC | AG | CC | AG | GG |  |  | AA | AG |  |
| CTRL | AA | CC | AA | TT | CC | GG | GG | GG | CC | CC | GG | CC | GG | GG |  |  | AA | AA |  |
| CTRL | AA | CC | AA | TT | CC | GG | GG | GG | CC | CC | GG |  | GG | GG |  |  | AG | AA |  |
| CTRL | AA | CC | AA |  | CC | GG | GG | GG | CC | CC | GG | CC | GG | GG |  |  | AA | AA |  |
| CTRL | GG | TT | CC | GG | TT | AA | CC | AA | TT | TT | AA | GG | AA | TT |  |  | AA | GG |  |
| CTRL | AA | CC | AA | TT | CC | GG | GG | GG | CC | CC | GG | CC | GG | GG |  |  | GG | AA |  |
| CTRL | AA | CC | AA | TT | CC | GG | GG | GG | CC | CC | GG | CC | GG | GG |  |  | AG | AA |  |
| CTRL | GG | TT | CC | GG | TT | AA | CC | AA | TT | TT | AA | GG | AA | TT |  |  | AA | GG |  |
| CTRL | AG | CT | AC | GT | CT | AG | CG | AG | CT | CT | AG | CG | AG | TT |  |  | AG | AG |  |
| CTRL | AA | CC | AA | GT | CC | GG | GG | GG | CC | CC | AA | CC | AA | GG |  |  | GG | GG |  |
| CTRL | GG | TT | CC | GG | TT | AA | CC | AA | TT | TT | AA | GG | AA | GT |  |  | AG | GG |  |
| CTRL | GG | TT | CC | GG | TT | AA | CC | AA | TT | TT | AA | GG | AA | GT |  |  | AA | GG |  |
| CTRL | AA | CC | AA | GT | CC | GG | GG | GG | CC | CC | AG | CC | AG | GG |  |  | GG | AG |  |
| CTRL | AA | CC | AA | TT | CC | GG | GG | GG | CC | CC | AG | CC | AG | GG |  |  | AG | AG |  |
| CTRL | AG | CT | AC | GT | CT | AG | CG | AG | CT | CT | AG | CG | AG | GT |  |  | AG | AG |  |
| CTRL | AG | CT | AC | GT | CT | AG | CG | AG | CT | CT | AG | CG | AG | GT |  |  | AG | AG |  |
| CTRL | AA | CC | AA | TT | CC | GG | GG | GG | CC | CC | AG | CC | AG | GG |  |  | AG | AG |  |
| CTRL | AA | CC | AA | TT | CC | GG | GG | GG | CC | CC | GG | CC | GG | GT |  |  | AG | AA |  |
| CTRL | AA | CC | AA | TT | CC | GG | GG | GG | CC | CC | AG | CC | AG | GG |  |  | GG | AG |  |
| CTRL | AA | CC | AA | TT | CC | GG | GG | GG | CC | CC | GG | CC | GG | GT |  |  | GG | AA |  |
| CTRL | AG | CT | AC | GT | CT | AG | CG | AG | CT | CT | AA | CG | AA | GT |  |  | GG | GG |  |
| CTRL | AA | CC | AA | TT | CC | GG | GG | GG | CC | CC | GG | CC | GG | GG |  |  | GG | AA |  |
| CTRL | AA | CC | AA | TT | CC | GG | GG | GG | CC | CC | AG | CC | AG | GT |  |  | GG | AG |  |
| CTRL | AA | CC | AA | TT | CC | GG | GG | GG | CC | CC | GG | CC | GG | GG |  |  | AG | AA |  |
| CTRL | AA | CC | AA | TT | CC | GG | GG | GG | CC | CC | GG | CC | GG | GT |  |  | AG | AA |  |
| CTRL | AA | CC | AA | TT | CC | GG | GG | GG | CC | CC | GG | CC | GG | GT |  |  | AA | AA |  |
| CTRL | AA | CC | AA | TT | CC | GG | GG | GG | CC | CC | GG | CC | GG | GG |  |  | AG | AA |  |
| CTRL | AA | CC | AA | TT | CC | GG | GG | GG | CC | CC | AG | CC | AG | GT |  |  | GG | AG |  |
| CTRL | AA | CC | AA | TT | CC | GG | GG | GG | CC | CC | GG | CC | GG | GT |  |  | GG | AA |  |
| CTRL | AA | CC | AA | TT | CC | GG | GG | GG | CC | CC | GG | CC | GG | GG |  |  | GG | AA |  |
| CTRL | AA | CC | AC | TT | CC | GG | CG | GG | CC | CC | GG | CG | GG | GG |  |  | AA | AG |  |
| CTRL | AA | CC | AA | TT | CC | GG | GG | GG | CC | CC | GG | CC | GG | GT |  |  | GG | AA |  |
| CTRL | GG | TT | CC | GG | TT | AA | CC | AA | TT | TT | AA | GG | AA | GT |  |  | AG | GG |  |
| CTRL | AA | CC | AC | TT | CC | GG | CG | GG | CC | CC | GG | CG | GG | GG |  |  | AG | AG |  |
| CTRL | AA | CC | AA | TT | CC | GG | GG | GG | CC | CC | GG | CC | GG | GT |  |  | AG | AA |  |
| CTRL | AA | CC | AA | TT | CC | GG | GG | GG | CC | CC | AG | CC | AG | TT |  |  | AG | AG |  |
| CTRL | AA | CC | AA | TT | CC | GG | GG | GG | CC | CC | GG | CC | GG | GG |  |  | AG | AA |  |
| CTRL | AG | CT | AA | GT | CT | AG | GG | AG | CT | CT | AG | CC | AG | GG |  |  | AG | AA |  |
| CTRL | AA | CC | AA | TT | CC | GG | GG | GG | CC | CC | GG | CC | GG | GG |  |  | AG | AA |  |
| CTRL | AA | CC | AA | TT | CC | GG | GG | GG | CC | CC | GG | CC | GG | GT |  |  | GG | AA |  |
| CTRL | AA | CC | AA | TT | CC | GG | GG | GG | CC | CC | GG | CC | GG | TT |  |  | AG | AA |  |
| CTRL | AA | CC | AA | TT | CC | GG | GG | GG | CC | CC | GG | CC | GG | TT |  |  | AG | AA |  |
| CTRL | AA | CC | AA | TT | CC | GG | GG | GG | CC | CC | GG | CC | GG | TT |  |  | AG | AA |  |
| CTRL | AA | CC | AA | TT | CC | GG | GG | GG | CC | CC | GG | CC | GG | GT |  |  | AA | AA |  |
| CTRL | AA | CC | AA | TT | CC | GG | GG | GG | CC | CC | AG | CC | AG | GT |  |  | AG | AG |  |
| CTRL | AA | CC | AC | TT | CC | GG | CG | GG | CC | CC | GG | CG | GG | GG |  |  | AA | AG |  |
| CTRL | AG | CT | AC | GT | CT | AG | CG | AG | CT | CT | AG | CG | AG | GT |  |  | AA | AG |  |
| CTRL | AA | CC | AA | TT | CC | GG | GG | GG | CC | CC | GG | CC | GG | GG |  |  | AG | AA |  |
| CTRL | AA | CT | AA | GT | CT | GG | GG | GG | CT | CT | AG | CC | AG | GG |  |  | AG | AA |  |
| CTRL | AG | CT | AA | GT | CT | AG | GG | AG | CT | CT | AG | CC | AG | GG |  |  | AG | AA |  |
| CTRL | AG | CT | AC | GT | CT | AG | CG | AG | CT | CT | AG | CG | AG | GT |  |  | GG | AG |  |
| CTRL | AA | CC | AA | TT | CC | GG | GG | GG | CC | CC | GG | CC | GG | GG |  |  | AG | AA |  |
| CTRL | AG | CT | AC | GT | CT | AG | CG | AG | CT | CT | AG | CG | AG | TT |  |  | AG | AG |  |
| CTRL | AA | CC | AA | TT | CC | GG | GG | GG | CC | CC | GG | CC | GG | GT |  |  | AG | AA |  |
| CTRL | AA | CC | AA | TT | CC | GG | GG | GG | CC | CC | GG | CC | GG | GG |  |  | GG | AA |  |
| CTRL | AA | CC | AA | TT | CC | GG | GG | GG | CC | CC |  | CC | GG | GT |  |  | AG | AA |  |
| CTRL | AA | CC | AA | TT | CC | GG | GG | GG | CC | CC | GG | CC | GG | GT |  |  | GG | AA |  |
| CTRL | AA | CC | AA | TT | CC | GG | GG | GG | CC | CC | GG | CC | GG | GG |  |  | AG | AA |  |
| CTRL | AA | CC | AA | TT | CC | GG | GG | GG | CC | CC | GG | CC | GG | GG |  |  | GG | AA |  |
| CTRL | AA | CC | AA | TT | CC | GG | GG | GG | CC | CC | GG | CC | GG | GG |  |  | AG | AA |  |
| CTRL | AA | CC | AA | GT | CC | GG | GG | GG | CC | CC | AG | CC | AG | GG |  |  | GG | AG |  |
| CTRL | AG | CT | AA | GT | CT | AG | GG | AG | CT | CT | AG | CC | AG | GG |  |  | AG | AA |  |
| CTRL | AA | CC | AA | TT | CC | GG | GG | GG | CC | CC | GG | CC | GG | GT |  |  | GG | AA |  |
| CTRL | AA | CC | AA | TT | CC | GG | GG | GG | CC | CC | GG | CC | GG | GT |  |  | AG | AA |  |
